# Mothers' intentions to discuss menstruation-related topics with their daughters using the theory of planned behavior

**DOI:** 10.11604/pamj.2025.51.52.46662

**Published:** 2025-06-19

**Authors:** Alemi Kebede Olika, Gurmesa Tura Debelew, Zewdie Birhanu Koricha, Muluemebet Abera Wordofa

**Affiliations:** 1 Institute of Health, Public Health Faculty, Population and Family Health Department, Jimma University, Jimma, Ethiopia,; 2 Institute of Health, Public Health Faculty, Department of Health Behavior and Society, Jimma University, Jimma, Ethiopia

**Keywords:** Intentions, mother-daughter communication, menstruation, theory of planned behaviour

## Abstract

**Introduction:**

there is a scarcity of research on mothers' intentions to engage in communication about menstruation-related topics with their daughters in Ethiopia. Therefore, the purpose of this study, which was based on the theory of planned behavior, was to investigate the characteristics that are linked to mothers' intentions to discuss menstruation-related topics with their daughters.

**Methods:**

between July 5-25, 2022, 390 mothers of adolescent girls aged 10 to 19 were selected as a sample and participated in the study.

**Results:**

a regression analysis revealed a strong association between mothers' intentions to discuss menstruation with their daughters and all three theory of planned behavior (TPB) components-attitudes, subjective norms, and self-efficacy. Self-efficacy was the factor that was significantly linked to greater intentions to discuss menstruation, followed by the attitude that mothers have about menstrual communication.

**Conclusion:**

these findings support the assumptions of the TPB as applied to mother-daughter menstrual health communication. These findings suggest that building self-efficacy and helping mothers feel responsible for educating their daughters about menstruation would be most important in attempting to increase mothers´ intentions to engage in mother-daughter communication on a wide variety of topics. Therefore, funding for parent-targeted programming should be allocated to increase parents' intentions to engage in mother-daughter communication. This programming should focus on helping mothers develop self-efficacy and a sense of responsibility for educating their daughters about multiple sexual and reproductive topics, including menstruation.

## Introduction

One possible source of information for adolescents and young people on sexual and reproductive health is their parents, and communication between parents and their children is one of the most important aspects of parenting [1-3]. Many positive outcomes in adolescent sexual health are associated with parent-child communication, including increased use of sexually transmitted infection (STI) and pregnancy prevention, increased self-efficacy in using contraceptives, and the capacity to negotiate for safe sex with a sexual partner [4-8]. Furthermore, effective menstrual communication gives adolescents the knowledge they need to make informed decisions, prevents unhygienic menstrual management practices, increases the likelihood of using safer menstrual management methods, and maintains good menstrual health [9,10].

Despite the advantages of parent-adolescent communication, parents may find it difficult to communicate effectively because of the sensitive nature of the topics involved, and talking to their children about managing their menstrual health can be challenging for parents [11]. Evidence from different research points toward the lack of mother-daughter communication about menstruation. According to data from Egypt, a little over one-third (35.0%) of moms talk to their daughters in order to get them ready for puberty [12]. Nearly two-thirds of mothers (65%) said they talked to their daughters about menstruation in Bihar, India [13]. A survey conducted in Northeast Ethiopia indicated that only 29.81% of respondents talked about menstruation and how to handle it [14].

The mother’s and daughter's lack of communication regarding menstruation can be attributed to several factors. Due to the taboo nature of menstruation, mothers are hesitant and ashamed to discuss it [15,16]. They assert that it is extremely challenging to have a conversation with the adolescent and that they are uncomfortable discussing such matters with their daughters [17-19]. As a result of their discomfort and embarrassment, mothers in five African countries confirmed that discussing menstrual issues with their daughters is shameful and prohibited [20]. Further, barriers come from parents´ internal processes, such as attitudes, subjective norms, and perception of self-efficacy, and their intention may prevent parents from engaging in successful parent and child communication. Thus, the theory of planned behaviour has been used in the field of parent-child communication around menstruation to investigate the mothers' intentions to discuss menstruation-related topics with their children. Ajzen (1991) asserts that a person's intentions have a significant role in determining their actual behaviour, and the possibility that a behaviour will be performed increases with an individual's level of intention.

Therefore, we hypothesised that communication between mothers and daughters on menstruation is dependent on the mother´s intention. The likelihood of mothers having a conversation with their daughter about menstruation increases with their intention to engage. Three factors-attitudes, subjective norms, and perceived behavioural control-influence this intention. Therefore, the study's objective was to examine predictors of mothers' intentions to discuss menstruation with their children. By identifying these mother-specific factors, it will be easier to understand why some parents choose to participate in mother-daughter menstrual communication while others do not. This will help educators and program developers better understand the areas that require education and intervention in an effort to increase mother-daughter menstrual communication and, in turn, improve the menstrual health of adolescents.

## Methods

**Study design, setting, and period:** a community-based cross-sectional survey was carried out from July 5-25, 2022, among mothers or female guardians of teenage girls between the ages of 10 and 19 in the Omo Nada and Shabe Sombo District of Jimma Zone, Oromia Regional State, Ethiopia.

**Source and study population:** the source populations were all mothers or female guardians of adolescent girls aged 10 to 19 years in the two selected districts. The study population consisted of randomly selected mothers or female guardians of adolescent girls aged 10 to 19 years who fulfilled the inclusion criteria.

**Sample size determination:** a single population proportion formula was used to get the sample size [21]:


$$n=\frac{Z^{2}pq}{d^{2}}$$

Where: n = desired sample size; Z = level of significance at 95% CI (=1.96); p = anticipated proportion of the mothers/caretakers who have intention to communicate about menstrual health and hygiene; q = 1-p and d = margin of error.

The following assumptions were taken into account: 50% of mothers have the intention to communicate about menstrual health because of a lack of previous studies that address the same objective, a 95% level of confidence, and a 5% margin of error. The total sample size, accounting for a 10% non-response rate, was 422.

**Sampling procedure:** respondents for the study were chosen using a simple random sampling technique. Initially, two districts-Omo Nada and Shabe Sombo were chosen at random. Secondly, 12 kebeles were chosen, with 6 coming from Omo Nada and 6 from Shabe Sombo. Thirdly, the lottery method was used to randomly select 15 Gares from each district. The process of choosing households was completed by first compiling a list of every household who have a mother/female guardian of adolescent girls aged 10 to 19 years in the chosen Gare, after which the required number of households was chosen at random, once more using the lottery technique. In the selected Gares, 13 houses were randomly selected. In order to be eligible for inclusion in the study, a mother or female guardian had to have a child between the ages of 10 and 19 years. In this study, a mother was defined as a child's biological mother or a female guardian who was not the child's biological parent but provided for the child's welfare.

**Data collection tool and procedure:** an elicitation study was conducted in the Kersa district prior to developing the data collection instrument in order to discover important beliefs that underlie attitudes, subjective norms, and perceived behavioral controls linked to mothers' menstrual health communication. The development of the questionnaire was informed by reviews of the literature of similar studies and advice gained from an elicitation study. The mothers were then asked to complete a structured, pre-tested questionnaire that was aided by an interviewer. Ten trained research assistants who were proficient in the local languages and had previous data collecting experience helped with the data collection, which was conducted in the local languages. Kobo Collect software was used to electronically collect the data.

**Data quality management:** initially, a structured questionnaire was developed in the English language and translated into Afaan Oromo and then back to English to maintain its consistency by experts. Then, the questionnaire was pretested with 20 mothers from the Kersa district to assess how easy it would be for respondents to understand the questions, and whether the questions measured the right constructs. The pre-test resulted in minor changes, such as the rephrasing of some questions to clarify their meaning. To further guarantee internal consistency, a reliability test was carried out. A Cronbach's alpha value of 0.7 or above was considered to be an acceptable level. Moreover, data quality was assured by conducting intensive supervision, and also checking submitted data on a daily basis. Statistical control during data analysis was used to reduce the influence of confounding factors.

**Variables:** the outcome variable was the mother´s intention towards menstrual health communication. The explanatory variables were the socio-demographic characteristics of the respondents and constructs of TPB: attitude, subjective norm, and perceived behavioural control.

**Measurements:** five Likert scale points were used to measure each of the TPB components (attitude, subjective norm, and perceived behavioral control) as well as behavioral intention toward menstrual health communication, with 1 representing strongly disagree, 2 disagree, 3 neutral, 4 agree, and 5 strongly agree. The responses received under each construct's components were added up to determine the variables.

**Intention toward menstrual health communication:** five items were used to measure intention. Intention was considered a continuous and categorical variable in this study. To find its predictors, intention was viewed as a continuous variable. The sum of the five items was used to get the overall intention score. A greater degree of intention to talk about menstruation is indicated by a higher score. The intention was divided into two categories according to the mean score in order to ascertain the prevalence. “Intended to have menstrual health communication” was defined as those who scored a mean score or higher, while “unintended to have menstrual health communication” was defined as those who scored below the mean.

**Attitudes toward menstrual health communication:** it describes the degree of favourability or unfavorability toward the desired behaviour. In order to assess respondents' attitudes regarding menstrual health communication, 24 items were used on a five-point Likert scale. Then, the sum of the scores was calculated; a higher score indicates a positive attitude toward menstruation health communication.

**Subjective norms:** this is the social component that shows whether respondents believed that significant people wanted them to talk about menstruation with their daughter. Fifteen items on a five-point Likert scale were used to measure this. The sum of the 15 items was then used to calculate the final score. The higher the score, the higher the approval of referents for mothers to communicate about menstruation.

**Perceived behavioural control:** another crucial factor is the perceived level of behavioral control. It describes a mother's assessment of how easy or hard it would be to discuss menstruation with her daughter. Fourteen items on a five-point Likert scale were used to measure it, and the sum of the items was used to determine the final score. A greater acceptance of discussing menstruation health is indicated by a higher score.

**Knowledge about mensuration:** mothers' knowledge was assessed by using 16 questions related to menstruation, out of which five were multiple-choice questions with three alternatives each. Eleven questions were true or false statements. One score will be allotted for each correct answer, and zero will be given for an incorrect answer. The possible range of obtainable scores will be between 0-16. The mean score is 10, and the variable was categorized as adequate knowledge for scores above the mean score and inadequate knowledge for less than the mean score.

**Data analysis:** Stata Version 17.0 was used to analyse the data. The analysis began with the computation of descriptive analysis such as mean, frequencies, and percentages. The correlations between the items were ascertained using Pearson's correlation coefficient. Predictors of mothers' intention toward menstruation health communication were found using univariate and multivariable linear regression analyses. First, univariate regression models were constructed using the mother's behavioural intention scores for each explanatory variable. After that, variables with a p-value less than 0.25 were analysed by fitting them into multivariable linear models. To find the independent effects of each explanatory variable on the outcome variable and account for confounding variables, multivariate linear regression analysis was used. Mothers' intention was shown to be significantly predicted by variables with p-values less than 0.05.

**Ethical approval:** the study was approved by the Institutional Review Board of Jimma University Institute of Health, with the reference number IHRPG1/541/21. The necessary permission was obtained from the Omo Nada and Shabe Sombo District Health Departments. Prior to data collection, eligible respondents were informed that the study aimed to understand their intentions to communicate about menstruation with their adolescents. Respondents were assured of anonymity and confidentiality of the collected data as well as the freedom to decline to participate at any time during data collection. Oral informed consent was obtained from all respondents.

## Results

**Sociodemographic characteristics:** in this study, a total of 390 mothers were incorporated. The mean age of the mother was 37.92 (±6.74) years. The Oromo ethnic group accounted for 85.64% of the participants, while the majority (84.36%) were from rural areas. The majority of respondents identified as Muslim religious followers. The majority of the mothers couldn't read or write, 262 (67.18%) were housewives, and 264 (72.53%) spouses couldn't read or write. Farming was the most common occupation for fathers; 306 (84.07%) were farmers. In terms of the size of the family, 308 people (78.97%) had five or more (Table 1).

**Table 1:** sociodemographic characteristics among mothers in Shabe and Omo Nada District, Jimma, Ethiopia, 2022

Variables	N (390)	Percentage
**Mother's mean age (mean and SD)**	**37.92 (±6.74)**	
**Place of residence**		
Urban	61	15.64
Rural	329	84.36
**Religion**		
Muslim	328	84.10
Orthodox Christian	50	12.82
Protestant Christian	12	3.08
**Ethnicity**		
Oromo	334	85.64
Amhara	40	10.26
Kafa	14	3.59
Other (Yem)	2	0.51
**Marital status**		
Married	358	91.79
Widowed	18	4.62
Others (single, separated, divorced)	14	3.59
**Education level**		
Unable to read and write	328	84.10
Informal education	34	8.72
Formal education	28	7.18
**Spouse’ education level**		
Unable to read and write	264	72.53
Informal education	62	17.03
Formal education	38	10.44
**Occupation**		
House wife	262	67.18
Farmer	104	26.67
Petty trade	18	4.62
Other (government employee and daily laborer)	6	1.54
**Spouse occupation**		
Farmer	306	84.07
Daily laborer	28	7.69
Merchant	20	5.49
Government employee	10	2.75
**Household family size**		
Small size (4 or fewer)	82	21.03
Large size (5 or more)	308	78.97
**Wealth index**		
Poorest	73	21.41
Poor	70	20.53
Medium	67	19.65
Rich	70	20.53
Richest	61	17.89
**Knowledge about menstruation**		
Adequate knowledge	171	43.85
Inadequate knowledge	219	56.15

**Measures of constructs of TPB:** as shown in Table 2, 158 (41.04%) and 160 (41.56%) disagreed that most people who are important to them and their neighbors approved of their talking about menstruation topics with their daughter, respectively. About one fourth, 97 (25.19%) and 89 (23.12%) of the mothers agreed that their husband and religious advisor approved their communication about menstruation with their daughter, respectively. Two hundred and seventy-six (45.71%), 178 (46.23%), and 155 (40.26%) of the mothers agreed that health care providers, school teachers, and mothers living in urban areas approved of their communication about menstruation with their daughter, respectively.

**Table 2:** descriptive statistics for the theory of planned behavior (TPB) constructs-subjective norm among mothers in Shabe and Omo Nada District, Jimma, Ethiopia, 2022 (n = 385)

Items of subjective norm	Strongly disagree	Disagree	Neutral	Agree	Strongly agree
Most people who are important to you, like family and friends, approve of your talking about menstruation topics with your daughter	17(4.52)	158(41.04)	125(32.47)	62(16.10)	23(5.97)
Most people like you have talked about menstruation topics with their daughters	21(5.45)	155(40.26)	143(37.14)	45(11.69)	21(5.45)
Most of your friends have talked about menstruation topics with their daughters	23(5.97)	138(35.84)	148(38.44)	58(15.06)	18(4.68)
Your friends would approve of your talking about menstruation topics with your daughter	15(3.90)	142(36.88)	120(31.17)	89(23.12)	19(4.94)
Your sister would approve of your talking about menstruation topics with your daughter	15(3.90)	134(34.81)	119(30.91)	92(23.90)	25(6.49)
Your close friends would approve of your talking about menstruation topics with your daughter	15(3.90)	131(34.03)	123(31.95)	93(24.16)	23(5.97)
Your husband would approve of you talking about menstruation-related topics with your daughter	17(4.42)	137(35.58)	107(27.79)	97(25.19)	27(7.01)
Your priest or religious advisor would approve of your talking about menstruation topics with your daughter	12(3.12)	145(37.66)	113(29.35)	89(23.12)	26(6.75)
My close friends approve of my talk about menstruation with my daughter	14(3.64)	110(28.57)	145(37.66)	100(25.97)	16(4.16)
Educated mothers approve of my talk about menstruation with my daughter	1(0.26)	76(19.74)	133(34.55)	134(34.81)	41(10.65)
Mothers who live in urban areas approve of my talk about menstruation with my daughter	1(0.26)	56(14.55)	137(35.58)	155(40.26)	36(9.35)
My daughter's friend's mother approves of my talk about menstruation with my daughter	8(2.08)	76(19.74)	156(40.52)	123(31.95)	22(5.71)
The school teacher approves of my talk about menstruation with my daughter	3(0.78)	43(11.17)	117(30.39)	178(46.23)	44(11.43)
Health care providers approve of my talk about menstruation with my daughter	2(0.52)	29(7.53)	110(28.57)	176(45.71)	68(17.66)
My neighbor would approve of me talking about menstruation topics with my daughter	35(9.09)	160(41.56)	110(28.57)	67(17.40)	13(3.38)

As shown in Table 3, more than half, 198 (51.30%) and 202 (52.33%) of the mothers agreed that talking with their daughter about menstruation is important, and the communication would improve their management practices, respectively. Similarly, the majority, 208 (53.89%) and 217 (56.22%) of the mothers believed that having communication would reduce feelings scared about menstruation and improve understanding of menstruation for both mothers and daughters. On the other hand, 112 (29.02%), 94 (24.35%), and 69 (17.88%) of mothers agreed that they cannot talk to their daughters about menstruation because they feel embarrassed to do so, believed that their daughters already know about menstruation, and think that talking about menstruation is a taboo, respectively.

**Table 3:** descriptive statistics for the theory of planned behavior (TPB) constructs-attitude regarding menstrual health communication among mothers in Shabe Sombo and Omo Nada District, Jimma, Ethiopia, 2022 (n = 386)

Items of attitude	Strongly disagree	Disagree	Neutral	Agree	Strongly agree
It is important to me that I can talk about menstruation with my daughter	4(1.04)	36(9.33)	103(26.68)	198(51.30)	45(11.66)
I think communication will reduce the stress that my daughter has related to menstruation	2(0.52)	48(12.44)	87(22.54)	207(53.63)	42(10.88)
It is necessary to talk to my daughter about menstruation because I can give her a solution for the problem she faces associated with menstruation	10(2,59)	45(11.66)	100(25.91)	194(50.26)	37(9.59)
I think talking about menstruation is important to know about things that we do not know about menstruation	4(1.04)	28(7.25)	103(26.68)	201(52.07)	50(12.95)
It is important to talk about menstruation because I can support my daughter during menstruation with anything she needs	3(0.78)	22(5.70)	92(23.83)	218(56.48)	51(13.21)
I think talking about menstruation to my daughter is important to know whether she is pregnant or not	3(0.78)	23(5.96)	96(24.87)	209(54.15)	55(14.25)
It is pleasurable to talk to my daughter about menstruation	7(1.81)	74(19.17)	102(26.41)	165(42.75)	38(9.84)
I think my daughter feels it is important that I can talk to her about menstruation	15(3.89)	63(16.32)	129(33.42)	158(40.93)	21(5.44)
If me and my daughter talk about menstruation, menstrual management practice of my daughter would be improved	2(0.52)	42(10.88)	98(25.39)	202(52.33)	42(10.88)
It is important to talk about menstruation to know whether my daughter is experiencing her menstruation or not menstruation	3(0.78)	23(5.96)	90(23.32)	219(56.74)	51(13.21)
If me and my daughter talk about menstruation, I will feel more positive, happier with myself, and feel more accomplished	4(1.04)	37(9.59)	105(27.20)	201(52.07)	39(10.10)
If me and my daughter talk about menstruation, it will reduce feeling ashamed and afraid of my daughter	1(0.26)	49(12.69)	89(23.06)	208(53.89)	39(10.10)
Myself and my daughter knowledge about menstruation will be improved if we talk	2(0.52)	29(7.51)	89(23.06)	217(56.22)	49(12.69)
I cannot talk with my daughter about menstruation because I feel embarrassed to do so	32(8.29)	130(33.68)	89(23.06)	112(29.02)	23(5.96)
If I would talk to my daughter about menstruation, I would not feel very comfortable	37(9.59)	155(40.16)	89(23.06)	91(23.58)	14(3.63)
I will feel bad if I communicate about menstruation with my daughter	44(11.40)	149(38.60)	87(22.54)	95(24.61)	44(11.40)
I cannot talk to my daughter about menstruation because I do not know enough about it	31(8.03)	156(40.41)	90(23.32)	92(23.83)	17(4.40)
I cannot talk about menstruation because my daughter is not likely to want to talk to me about menstruation	22(5.70)	155(40.16)	93(24.09)	89(23.06)	27(6.99)
It is not necessary to talk to my daughter about menstruation, because she already knows enough about it	22(5.70)	135(34.97)	109(28.24)	94(24.35)	26(6.74)
As my daughter did not face problems associated with menstruation, it is not important to talk with her	29(7.51)	162(41.97)	94(24.35)	81(20.98)	20(5.18)
I thought my daughter would feel embarrassed if I talked about menstruation with her, as a result I prefer to keep quiet	25(6.48)	145(37.56)	95(24.61)	94(24.35)	27(6.99)
It is not important to talk about menstruation to your daughter because it is a breach of respect between us	46(11.92)	173(44.82)	96(24.87)	56(14.51)	15(3.89)
I think I will lose time for other things if I talk about menstruation with my daughter	26(6.74)	183(47.41)	97(25.13)	69(17.88)	11(2.85)
It is not important to talk about menstruation since it is taboo	26(6.74)	183(47.41)	97(25.13)	69(17.88)	11(2.85)

Findings showed that the majority, almost half, 190 (49.35%) of the mothers agreed that they have enough information about menstruation that enables them to talk with their daughter. Similarly, the majority, 227 (58.96%) of the mothers agreed that they were able to answer the question being asked by their daughter about menstruation. However, for about one fourth (93 (24.16%)) of the mothers, they agreed that they are uncomfortable talking about menstruation, whereas 75 (19.48%) and 87 (22.60%) of the mothers agreed that they find it difficult and hard to talk with their daughter about menstruation, respectively (Table 4).

**Table 4:** descriptive statistics for the theory of planned behavior (TPB) constructs-perceived behavioural control among mothers in Shabe and Omo Nada District, Jimma, Ethiopia, 2022 (n = 385)

Items of perceived behavioural control	Strongly disagree	Disagree	Neutral	Agree	Strongly agree
I have enough information about menstruation issues to talk to my daughter	7(1.82)	37(9.61)	122(31.69)	190(49.35)	29(7.53)
If my daughter asked me a question about a menstruation issue, I would respond	7(1.82)	20(5.19)	94(24.42)	220(57.14)	44(11.43)
I can answer the questions my daughter has about menstruation	9(2.34)	16(4.16)	90(23.38)	227(58.96)	43(11.17)
I am free to talk to my child about menstruation-related issues	10(2.60)	72(18.70)	98(25.45)	171(44.42)	34(8.83)
My daughter and I can talk openly (frankly) and freely about menstruation	26(6.75)	92(23.90)	116(30.13)	134(34.81)	17(4.42)
I can answer questions that my daughter asks me about menstruation frankly and honestly	13(3.38)	33(8.57)	114(29.61)	194(50.39)	31(8.05)
If my child asked me a question about a menstruation issue, I would be embarrassed	16(4.16)	78(20.26)	85(22.08)	172(44.68)	34(8.83)
When I talk to my child about menstruation, I tell her about the negative things about menstruation	32(8.31)	195(50.65)	98(25.45)	50(12.99)	10(2.60)
I am uncomfortable if my child asks me a question about a menstruation issue	12(3.12)	67(17.40)	94(24.42)	172(44.68)	40(10.39)
It is hard for me to talk with my daughter about menstruation	16(4.16)	169(43.90)	90(23.38)	87(22.60)	23(5.97)
My daughter does not allow me to ask them questions about menstruation	11(2.86)	136(35.32)	127(32.99)	86(22.34)	25(6.49)
I find it difficult talking to my daughter about menstruation	17(4.42)	165(42.86)	103(26.75)	75(19.48)	25(6.49)
I am uncomfortable talking about menstruation with my daughter because it is taboo	37(9.61)	140(36.36)	97(25.19)	93(24.16)	18(4.68)
As my daughter feels embarrassed to talk with me, I also feel the same to talk with her	31(8.05)	162(42.08)	88(22.86)	81(21.04)	23(5.97)

**Intention toward menstrual health communication:** the majority, 166 (43.12%) of the mothers agreed with the question that asked I plan to talk about menstruation-related topics with my daughter within the next six months. Similarly, 161 (41.82%) of mothers have a plan to talk about how their daughter could manage her menstrual bleeding within the next six months. The study also found that 220 (57.15%) of them intended to talk about menstruation with their daughters in the following six months (Table 5).

**Table 5:** descriptive statistics for the theory of planned behavior (TPB) constructs-intentions among mothers in Shabe and Omo Nada District, Jimma, Ethiopia, 2022 (n = 385)

Items of intention	Strongly disagree	Disagree	Neutral	Agree	Strongly agree
I plan to talk about the menstruation-related topic with my daughter within the next six months	20(5.19)	131(34.03)	35(9.09)	166(43.12)	33(8.57)
I plan to talk with my daughter in the next six months about cultural beliefs related to menstruation	17(4.42)	126(32.73)	43(11.17)	172(44.68)	27(7.01)
I plan to talk about how my daughter could manage her menstrual bleeding next six months	21(5.45)	118(30.65)	47(12.21)	161(41.82)	38(9.87)
I plan to talk about guidance on how to dispose of menstrual products next six months	15(3.90)	128(33.25)	46(11.95)	156(40.52)	40(10.39)
I plan to talk about supporting my daughter's mental and emotional health during menstruation next six months	18(4.68)	123(31.95)	44(11.43)	164(42.60)	36(9.35)
**Intention toward menstrual health communication**	**Number**	**Percentage**			
Intended to have communication	220	57.15			
Unintended to have communication	165	42.85			

**Mean scores of the constructs of TPB and the correlation analysis result:** a descriptive statistical analysis was done to measure the mean score of TPB components. Attitude, subjective norm, and perceived behavioural control had a mean score of 82.71 (SD = 12.84), 46.52 (SD = 9.52), and 45.45 (SD = 7.33), respectively. The mean score of intention was 15.29 (SD = 3.33) (Table 6).

**Table 6:** descriptive statistics for the components of the theory of planned behaviour model, Pearson’s correlation between components of the theory of planned behavior (TPB) model and mothers’ intention in the selected district, Jimma, Ethiopia, 2022 (n=385)

Components	No of items	Min. value	Max. value	Mean (95%CI)	SD
Attitude	24	33	120	82.71(81.37 - 83.94)	12.84
Subjective norm	15	23	75	46.52(45.57- 47.48)	9.52
Perceived behavioural control	14	18	66	45.45(44.72-46.19)	7.33
Intention	5	6	24	15.29(14.96-15.62)	3.33
**Pearson’s correlation between components of the TPB model**					
**Components**	**Attitude**	**Subjective norm**	**Perceived behavioural control**	**Intention**	
Attitude	1				
Subjective norm	0.464*	1			
Perceived behavioural control	0.713*	0.326*	1		
Intention	0.577*	0.375*	0.558*	1	

* : correlation is significant at the 0.05 level

A Pearson´s correlation analysis was done to examine the association between components of the TPB model and mothers´ intention towards menstrual health communication. The findings indicated that there was a positive and moderate correlation between attitude and subjective norm. Attitudes were also seen to have a moderate level of association with the intentions of the mothers to have communication about menstruation with their daughter. Subjective norms had a moderate level of association with the perceived behavioural control and intentions of the mothers to have communication about menstruation with their children. Perceived behavioural control had the strongest association with the attitude of the mother to have communication about menstruation with their daughter (Table 6).

**Predictors of mothers´ intention towards menstrual health communication:** simple linear regression analysis was conducted to assess the association between intention and constructs of TPB. All the constructs and sociodemographic variables were candidate variables from simple linear regression analysis and entered into multiple linear regression analysis. After controlling for confounding factors, subjective norm, perceived behavioural controls, and attitude were predictors of mothers´ intention to communicate with their daughters about menstrual health. For a positive unit change in the attitude towards menstrual health communication, intention to communicate was increased by 0.072 units (adjusted β = 0.072; 95% CI 0.035 to 0.109; P < 0.00), provided that other variables were kept constant. Mothers' social network determines their intention toward menstrual health communication. For a positive unit change in social networks´ approval of menstrual health communication, mothers´ intention toward communication was increased by 0.057 units (adjusted β = 0.057; 95% CI 0.022 to 0.092; P = 0.002) given that other variables were kept constant. For a positive unit change in perceived behavioural control to communicate about menstrual health, the intention was increased by 0.124 units (adjusted β = 0.124; 95% CI 0.064 to 0.185; P < 0.00) provided that other variables are kept constant (Table 7).

**Table 7:** independent factors associated with mothers' behavioural intention to menstrual health communication, Jimma, Ethiopia, 2022

Variables	Mothers' behavioural intention towards Menstrual health communication		
	β	95% [CI]	P-value
Constant	2.202	[-1.275 to 5.680]	0.214
Perceived behavioural control	0.124	[0.064 to 0.185]	<0.001
Subjective norm	0.057	[0.022 to 0.092]	0.002
Attitude	0.072	[0.035 to 0.109]	<0.001
Age	-0.046	[-0.092 to 0.000]	0.051
**Place of residence**			
Urban	1.133	[0.040 to 2.227]	0.042
Rural	Ref.		
**Education level**			
Unable to read and write	Ref		
Informal education	-0.190	[-1.304 to 0.924]	0.737
Formal education	-0.371	[-1.749 to 1.008]	0.597
**Household family size**			
Small size (4 or fewer)	Ref.		
Large size (5 or more)	-0.832	[-1.684 to 0.020]	0.056
**Knowledge about menstruation**			
Adequate knowledge	-0.040	[-0.667 to 0.587]	0.900
Inadequate knowledge	Ref.		
**Wealth index**			
Poorest	Ref.		
Poor	0.427	[-0.495 to 1.349]	0.363
Medium	-0.089	[-1.033 to 0.855]	0.853
Rich	-0.300	[-1.251 to 0.651]	0.535
Richest	0.059	[-1.088 to 1.208]	0.918
**Spouse’s education level**			
Unable to read and write	Ref.		
Informal education	0.214	[-0.697 to 1.124]	0.644
Formal education	0.554	[0.609 to 1.712]	0.350
**Occupation**			
House wife	Ref.		
Farmer	-0.177	[-0.882 to 0.528]	0.622
Others (petty trade, gov’t employee, and daily labourer)	0.115	[-1.511 to 1.742]	0.889
**Spouse occupation**			
Daily l labourer	Ref.		
Farmer	0.959	[-0.463 to 2.381]	0.185
Government employee	0.692	[-1.552 to 2.936]	0.544
Merchant	0.542	[-1.252 to 2.336]	0.553

## Discussion

Mothers can play an important role in children´s education at home, and gaining an understanding of the determinants of mothers´ intentions to communicate about menstruation with their children is critical for developing and implementing programmes. This study investigated the determinants of mothers´ intention to communicate about menstruation and used TPB to explain intention to communicate. The correlation analysis showed that attitude and perceived behavioural control towards menstrual health communication were the strongest correlates of mothers´ intentions to communicate, followed by subjective norms. The regression analysis also showed that attitudes, subjective norms, and perceived behavioural control towards menstrual health communication best predicted mothers´ intention to communicate about menstruation. The results of this study support the theory of planned behaviour as attitudes, subjective norms, and perceived behavioural control were significantly associated with the intentions of the mothers to have communication about menstrual health with their daughters [22].

Attitudes have a significant level of association with the intentions of the mothers to have communication about menstruation with their children. The majority of mothers in this survey recognize the value and benefits of communication, as evidenced by the fact that over 50% of them think that communication enhances management practices and lessens feelings of shame related to menstruation. This suggests that intentions to discuss basic biological topics could be determined more by mothers´ beliefs that they understand the worth of the issue and are responsible for providing their child with adequate information. Given that mothers play an important role as gatekeepers for their children, a favourable mother attitude towards menstrual health communication is a key in determining mothers´ intentions to communicate. Interventions aimed at influencing mothers´ intentions to communicate about menstrual health should therefore consider influencing mothers' attitudes towards communication as well.

The present study also showed a significant association between perceived social network pressure and mothers´ intention toward communication about menstruation with their children. The intention of an individual to engage in a specific behaviour is influenced by whether or not their social system accepts or rejects it, and the respondents perceived that their social networks think they need to talk about menstruation with their daughter. This is consistent with the study done by Astle *et al*. (2022) and Khandelwal *et al*. (2022) that clearly showed that subjective norms were seen to be the significant predictor of intentions of having parent-child sexual communication [23,24]. This study shows the powerful influence of social networks on mothers´ decisions regarding the communication about menstruation with their daughters. Given the influence of referent groups on mothers´ intentions, menstrual health programme planners should consider consulting the influential referent groups in the development of programmes. Subjective norms can be increased among mothers by organizing community programs where community members can help each other in a variety of ways like helping mothers who are not sure how to engage in conversation with their children, increasing awareness and importance of communication about menstruation, sharing some stories and experiences, encouraging each other, etc.

Further, the results of the study reveal perceived behavioural control to be the most significant variable in increasing the intentions of the parents to have communication about menstruation with their daughter. This suggests that parents´ own feelings of self-efficacy in their ability to discuss menstrual-related topics with their children are a facilitator of intending to engage in these conversations with children. This finding was consistent with the study from India that indicated that perceived behaviour control is the most significant variable related to intentions of parent-child communication about sexuality [24]. Therefore, efforts should be made to increase the perceived behavioural control of the parents with the help of various programs, training, and mother education. These programs should focus not only on increasing the knowledge and awareness of the mothers but also on increasing the confidence and motivation of the mothers to engage in the conversations.

Despite the fact that it had significant implications and strengths, such as applying the theory of planned behaviour concept to determine the predictors of mothers' intention regarding menstruation and measuring the issues that were not present in the literature. Limitations should be considered when interpreting the results of this study. Even though measuring mothers' intentions regarding menstrual health communication offers valuable insight into what mothers are planning, the results of this study should only be applied to mothers' intentions and not to actual mother-daughter communication regarding menstruation.

## Conclusion

From this study, it was understood that the predictors of mothers´ behavioural intention to talk about menstruation with their daughters were attitude, subjective norm, and perceived behavioural control. Adequate and appropriate information about menstrual health is critical for the overall development of adolescent girls, so it is important that mothers and adolescent girls are able to communicate about menstrual health issues in a proper manner. These results have significant practical implications, especially for parent education. First, these findings imply that boosting mothers' self-efficacy and assisting them in creating favourable attitudes and norms about mother-daughter communication should be the main goals of removing obstacles to mothers' intentions to communicate with their daughters. Giving parents information about many menstruation-related subjects might help them feel secure in their knowledge and help them develop self-efficacy. The findings demonstrated that interventions to influence mothers to support communication about menstruation may not be successful if they fail to engage with social norms and beliefs regarding the importance of communication. Therefore, increasing subjective norms should continue to be taken into consideration when developing and implementing mother-daughter communication programs because of their significant association with mother-daughter menstrual communication intentions. Creating support groups for parents who want to talk about their own mother-daughter communication experiences could be one way to do this.

### What is known about this topic

- Menstruation has been bound by silence and is frequently absent from daily conversations;
- This culture of silence around menstruation causes a substantial number of girls to have inadequate knowledge and be unaware of what menstruation is before they experience their first menses;
- Parent-adolescent communication is one of the potential sources of information and protective factors for adolescent development.

### What this study adds

- Research about mothers´ intention to discuss menstruation-related subjects with their daughters is lacking in Ethiopia, and this study highlighted the importance of mothers´ intention in influencing mother-daughter communication about menstruation;
- Findings suggest that the primary objectives of reducing barriers to mothers' intentions to interact with their daughters should be to increase mothers' self-efficacy and help them establish positive attitudes and norms regarding mother-daughter communication.
